# Dry Needling Produces Mild Injuries Irrespective to Muscle Stiffness and Tension in Ex Vivo Mice Muscles

**DOI:** 10.1155/2022/8920252

**Published:** 2022-07-05

**Authors:** Marc Bosque, Ramon Margalef, Oscar Carvajal, David Álvarez, Manel M. Santafe

**Affiliations:** ^1^Unit of Histology and Neurobiology, Department of Basic Medical Sciences, Faculty of Medicine and Health Sciences, Rovira i Virgili University, Reus, Spain; ^2^Clínica Fisioterapia Océano, Servicio de Fisioterapia, Calle Las Palmas, 55, 28938 Móstoles, Madrid, Spain

## Abstract

Numerous studies have suggested that the myofascial trigger points are responsible for most of the myofascial pain syndrome, so it seems reasonable that its destruction is a good therapeutic solution. The effectiveness of dry needling (DN) has been confirmed in muscles with myofascial trigger points, hypertonicity, and spasticity. The objective of this study is to analyze the need of repetitive punctures on muscles in different situations. The levator auris longus (LAL) muscle and gastrocnemius muscle from adult male Swiss mice were dissected and maintained alive, while being submerged in an oxygenated Ringer's solution. DN was evaluated under four animal models, mimicking the human condition: normal healthy muscles, muscle fibers with contraction knots, muscles submerged in a depolarizing Ringer solution (KCl-CaCl_2_), and muscles submerged in Ringer solution with formalin. Thereafter, samples were evaluated with optical microscopy (LAL) and scanning electron microscopy (gastrocnemius). Healthy muscles allowed the penetration of needles between fibers with minimal injuries. In muscles with contraction knots, the needle separated many muscle fibers, and several others were injured, while blood vessels and intramuscular nerves were mostly not injured. Muscles submerged in a depolarizing solution inducing sustained contraction showed more injured muscular fibers and several muscle fibers separated by the needle. Finally, the muscles submerged in Ringer solution with formalin showed a few number of injured muscular fibers and abundant muscle fibers separated by the needle. Scanning electron microscopy images confirm the optical analyses. In summary, dry needling is a technique that causes mild injury irrespective of the muscle tone.

## 1. Introduction

The most frequent cause of muscular pain is the myofascial pain syndrome (MPS) [1]. MPS is a collection of known sensory, motor, and autonomic symptoms caused by myofascial trigger points (MTrPs) [2, 3]. Myofascial trigger points are hyperirritable nodules within taut bands of skeletal muscle responsible for sensory, motor (stiffness, weakness, and restricted range of motion), and autonomic dysfunction [3]. Electromyography defines this as “spontaneous electrical activity” (SEA), also called “end plate noise.” The irritability of the MTrP can be objectively assessed in rabbit alongside the changes in prevalence or amplitude of SEA that are recorded in this region [4]. Histologically, in the mouse myocyte, it appears as a contraction knot located close to a synaptic contact, constituting what is called “active loci” (a well-defined area of accumulation of dysfunctional motor plates) [5].

The most common therapeutic technique employed in the treatment of MTrPs is dry needling (DN) [6]. DN consists of using the mechanical stimuli of a needle to either eliminate or inactivate the MTrP. The DN technique requires multiple insertions [7–9]. The number of needle insertions is associated with postneedling soreness [10]. In our laboratory, we repeatedly needled the levator auris longus muscle of mice and described denervation and fiber muscular damage [11]. These repeated muscle punctures do not interfere with the different stages of normal muscle regeneration and reinnervation. However, little is known about the local effects of repeated DN immediately after the puncture.

In subjects with MTrPs, DN has been reported to decrease pain [12]. The possible physiological effects of DN involve both mechanical stimulation and the consequences of local microtrauma. DN can influence the SEA by causing a local twitch reflex (LTR). LTR is an involuntary spinal reflex that results in a localized contraction of affected muscle fibers that are manually stretched with dry needles. Both Chen et al. [6] and Hsieh et al. [12] demonstrated in their studies with rabbit that DN to a MTrP region could effectively suppress SEA, when LTRs were elicited. They suggested that inserting a needle into the endplate region may lead to increased discharges and thus immediately reduce available ACh stores, leading to a decrease in the SEA. Another possible mechanism could be that sufficient mechanical activation of the puncture around the endplate area causes the muscle fibers to discharge, thereby producing a LTR. Baldry [13] mentioned that a LTR causes alterations in muscle fiber length and tension and stimulates mechanoreceptors such as A*β* fibers. On the other hand, microtrauma caused by needle insertion can increase blood flow and muscle oxygenation. Indeed, local microtrauma leads to the release of vasoactive substances, such as CGRP and SP, which are also increased after the activation of *C* fibers through the axon reflex, leading to vasodilation of small vessels [11]. The clinical and scientific evidence of the beneficial effects of DN has been on the increase over the years [14, 15]. But there is no consensus on how many inserts are needed. For example, Fernández-Carnero et al. [16] report that different numbers of DN (including not generating LTRs and generating six LTRs, or continuing until no more LTRs are obtained) in the upper trapezius produce similar improvements in patients with cervical myofascial pain. Moreover, the mechanisms of action of DN in managing other muscle tone abnormalities (such as hypertonia or spasticity) have been poorly studied. Nonpharmacological treatments like dry needling have been used for years to decrease hypertonia and spasticity (DNHS®, Dry Needling for Hypertonia and Spasticity) and improve the function of muscles [13]. Spasticity is a common disabling motor deficit after a stroke [17–19]. DN of the hypertonic and spastic muscles in individuals with stroke produces an immediate reduction in spasticity and an increase in active range of motion [8, 9]. Some studies have tried to objectively quantify the adverse effects of the DNHS technique on the contractile properties of spastic muscles [20], but there is still a dearth of knowledge regarding this subject.

Despite repeatedly inserting a solid needle into the muscles of patients, the role of DN in muscles with MTrPs, hypertonia, or spasticity is not well known. There are no biological foundations for multiple insertions. The working hypothesis of this study is that increased muscle stiffness and tension are associated with increased likelihood of muscle fiber injury from dry needling and, therefore, provide elements to decide the need for multiple insertions.

## 2. Materials and Methods

### 2.1. Animals and Muscles

Adult male Swiss mice (30 to 40 days of age; Charles River, L'Arbresle, France) were used for this study (15 animals). The animals were housed in plastic cages containing 2–5 individuals and allowed free access to food and water all through the period of experiment. The animals' room was maintained at a temperature of 22 ± 2°C, a relative humidity of 50 ± 10%, and a 12 h light/dark automatic light cycle. This study was approved by the Ethics Committee CEIm-IISPV (Ref. 178/2019). The mice were cared for in accordance with the guidelines of the European Community's Council Directive (2010/63/EU) for the humane treatment of laboratory animals.

Animals were sacrificed by exsanguination under anesthesia. Thereafter, the *levator auris longus* (LAL) muscle was dissected. This muscle was chosen because it is a thin and flat muscle with a well-known intramuscular nerve branching pattern and it is easy to handle, while applying specific techniques to obtain optical images that allow us to observe the whole fiber and the contraction knot [5]. Recently, in our laboratory, we created an animal model with myofascial trigger points produced by a single subcutaneous injection of neostigmine [5]. Neostigmine induces an increase in spontaneous ACh release, followed by a cascade of events that finally results in contraction knots.

### 2.2. Dry Needling

The animals were anesthetized with 2% tribromoethanol (0.15 ml/10 g of body weight, I.P.). The LAL and gastrocnemius muscles were extracted and pinned on Sylgard-coated Petri dish containing normal Ringer solution (containing (in mM): 135 NaCl, 5KCl, 2.5 CaCl_2_, 1 MgSO4, 1 NaH_2_PO_4_, 15 NaHCO_3_, and 11 glucose) continuously bubbled with 95% O_2_/5% CO_2_. Thereafter, repeated punctures on the muscles were performed. Muscles immersion in oxygenated normal Ringer is a conventional procedure when some aspects of muscle physiology are to be studied. Upon these conditions, the muscle maintains the ability to contract and react to the physical aggression of inserting a solid needle. In this study, we used a solid needle habitually used for acupuncture and dry needling (0.25 mm thick and 25 mm long; AguPunt, Barcelona, Spain). Punctures were made at different sites in the muscle. The LAL muscle is a flat muscle, so when a puncture is made, a hole remains. In this experimental condition, if that hole is punctured again, the rest of the muscle fibers remain unaffected. Furthermore, dry needling has also been performed in the gastrocnemius muscle since this muscle is thicker than the LAL. Under the conditions of performing dry needling (punctures in different sites of the muscle), not more than 15 punctures on the LAL muscle can be performed. In both the LAL and the gastrocnemius, all punctures were performed in the middle third of the muscle, where the synaptic contacts are concentrated. To mimic muscle conditions in human patients, four types of situations have been designed (from low to high muscle contraction): normal healthy muscles; muscles with contraction knots (mimicking human muscles with MTrPs; thirty minutes after a subcutaneous administration of neostigmine; for more details, see Margalef et al. [5]); muscles submerged in a depolarizing Ringer solution (KCl, 20 mM) rich in calcium (CaCl_2_, 5 mM) mimicking human muscles with hypertonia; and muscles submerged in a Ringer solution with formalin (4%) mimicking spastic muscles. Treatment with neostigmine causes the subsynaptic contraction knots to appear in muscle fibers. Muscle fibers with contraction knots cause shortening of the sarcomeres in the area where the knots are. However, it has been described that the rest of the fiber is tighter [21]. The depolarizing Ringer solution rich in calcium sustains contraction throughout the entire length of the muscle fiber. The action of formalin on living muscle fibers is to achieve the highest homogeneous contraction throughout their length (like that caused in spasticity). Muscle samples were studied with optical microscopy and scanning electron microscopy. Fifteen animals per group were used (total 60 animals). In almost all groups, the muscle has been dissected and immersed in different types of physiological solutions. The group treated with neostigmine was anesthetized and injected subcutaneously with neostigmine and 30 minutes after the muscle was extracted and immersed in Ringer to perform the punctures. Of the initial 15 animals in neostigmine group, 2 died. Something similar happened with the samples mimicking spasticity. Once extracted, the muscle was pinned and immersed in a formalin solution. The formalin-induced contraction was so significant that 6 muscles were torn from their pins and had to be discarded from the study.

### 2.3. Methylene Blue Staining

Only LAL muscles were processed for optical study and morphometric analyses. The LAL muscles were fixed in 10% neutral formalin for 3 to 10 days and exposed to a 1% methylene blue dissolved in 1% borax for two minutes. Subsequently, the samples were washed with distilled water for two minutes each during the three steps. Finally, we proceeded to dehydration and mounting with epoxy resin.

The samples were observed at 1000x with an optical microscope to evaluate the number of injured fibers per hole. An injured fiber is considered when it is seen to be cut and/or dilated. The LAL is an extremely flat muscle, so it was easy to visualize these fibers surrounded by a hole created by the insertion of the needle. The number of fibers injured per hole created by the insertion of the needle has been counted. The experimental unit is the muscle hole. Fifteen insertions were made, which are the maximum that the muscle admits. In this sense, there was no randomization.

### 2.4. Scanning Electron Microscopy (SEM)

Only gastrocnemius muscles treated with neostigmine were processed for SEM observations. This muscle was chosen because of its thickness and the presence of abundant connective tissue surrounding its fibers. The connective tissue that surrounds the fibers of the LAL muscle is not very evident, unlike in the gastrocnemius muscle. The connective tissue contributes to the stability of muscle tissue. For this reason, the samples have been processed without using collagenase. All samples were dehydrated in sequence with increasing alcohol concentrations (50%, 70%, 80%, 90%, and 100%; V alcohol/V demineralized water) and then dried at room temperature. Samples were observed using an environmental scanning electron microscopy (FEI ESEM Quanta 600 FEG—Environmental Scanning Electron Microscope, Graz, Germany). In addition, needle tips (0.25 mm thick and 25 mm long; AguPunt, Barcelona Spain) were visualized with the SEM.

### 2.5. Statistical Procedure

Data were analyzed using SPSS version 21.0 (SPSS, Inc., Chicago, IL, USA). Results are expressed as means ± standard deviation (SD), considering the 95% CI. Normality was assessed by Shapiro–Wilk test, the Kolmogorov–Smirnov test was used for comparisons between groups not showing a normal distribution, and differences were considered significant at *P* < 0.05.

## 3. Results

### 3.1. DN in Healthy Muscles

**Figure 1:**
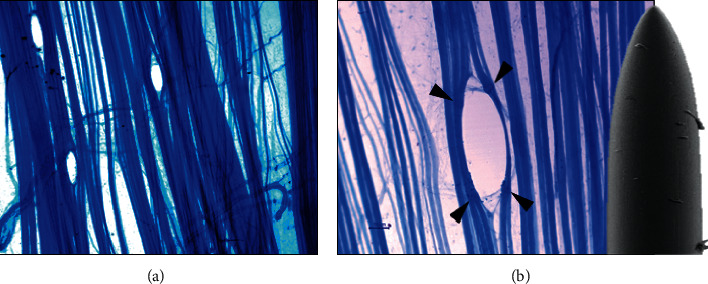
Ex vivo dry needling with solid needle in normal healthy LAL muscle. (a) Three punctures were performed in the innervation area of the muscle. Note that few muscle fibers are injured, and the needle sets many of them a side (initial magnification: 100x). (b) Detail of a puncture. The SEM image is an example of a solid needle habitually used for acupuncture and dry needling (diameter of 250 *μ*m). Arrowheads, muscle fiber injury points. Two images are on the same scale (scale bar: 50 *μ*m).

As given in Table 1, the number of injured fibers was very low in healthy muscles (about 4).  Figure 1 shows how there are almost no muscle fibers sectioned and most have been set aside. The punctures were made in the innervation band. In Figure 1(a), several intramuscular nerves can be seen to cross the image.  Figure 1(b) shows a greater magnification and how some fibers were injured, and others were separated by the needle. Moreover, Figure 1(b) shows the use of a solid needle for insertions observed using the scanning electron microscopy. The magnifications of the methylene blue image and the needle are the same. Note that the hole in the puncture zone is smaller than the diameter of the needle. When the needle is removed, the muscle fibers return to their initial position, partially closing the hole created.

### 3.2. DN in Muscles with Fibers with Contraction Knots

**Figure 2:**
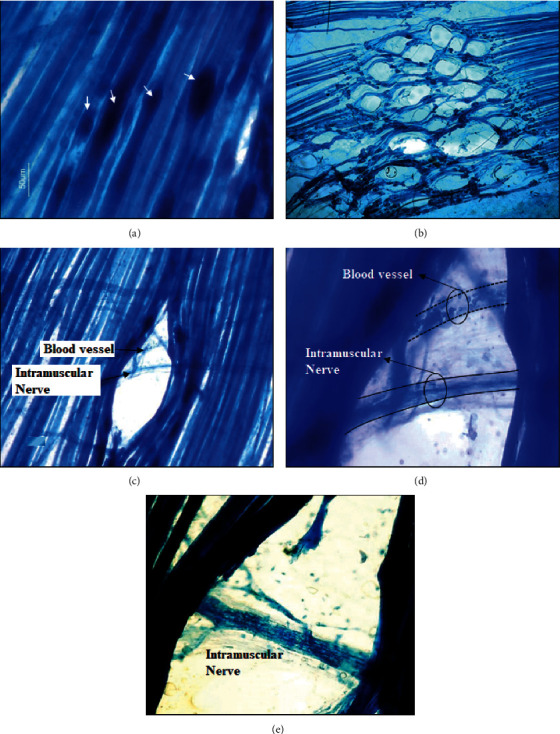
Ex vivo dry needling in LAL muscle with fibers with contraction knots. (a) Thirty minutes after a subcutaneous administration of neostigmine, the muscle shows several knots of contraction (arrows). (b) Several repeated punctures on the muscle were performed. Note that punctures were not performed at the same point. The needle separates many muscle fibers, and many others are injured (initial magnification: 100x). (c) Details of a puncture. Vessels and nerves in the puncture site can be seen without injury (initial magnification: 200x). Erythrocytes circulating through the vessel can be seen (d) (initial magnification: 400x). (e) Another example of puncture showing an intramuscular nerve without injury (initial magnification: 400x).

Thirty minutes after a subcutaneous administration of neostigmine, several knots of contraction can be observed (Figure 2(a)). We observed that there are also lateral displacements and lower injuries than expected (Figure 2(b)). The number of fibers injured by the needle increases by 10% with increasing tension in these muscles and fibers with contraction knots (Table 1).  Figure 2(c) shows how the needle separates some muscle fibers and some are injured in the process. In this kind of experiments, the punctures were also made in the innervation band. Surprisingly, in many cases, the tip of the needle also separates structures such as nerves or blood vessels (Figures 2(c) and 2(d)). In Figure 2(c), erythrocytes remain in the undamaged blood vessel. In areas of puncture, intact intramuscular nerves could also be seen (Figure 2(d)).

### 3.3. DN in Muscles Treated with KCl/CaCl_2_

**Figure 3:**
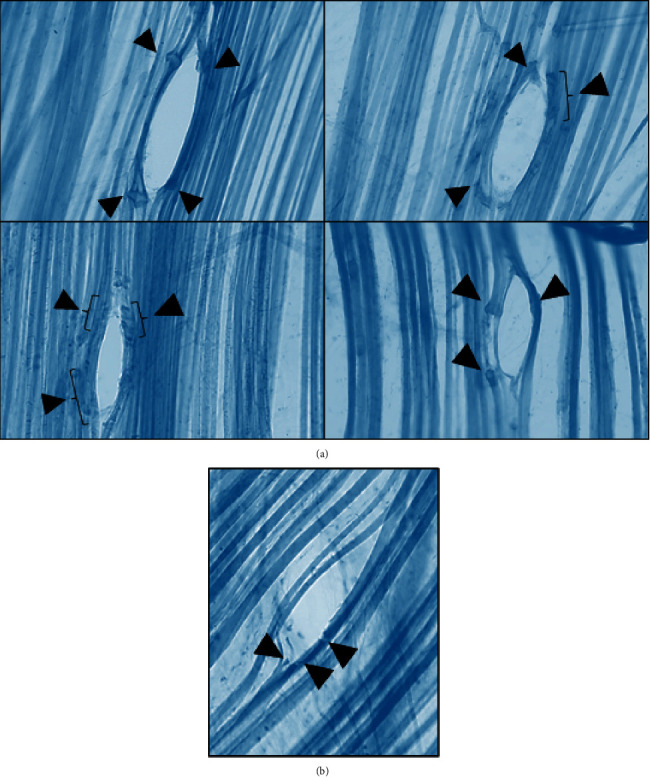
Ex vivo dry needling in contracted healthy LAL muscle. (a) Muscles were contracted with a calcium-rich depolarizing medium (KCl/CaCl_2_). In the different examples, more injured fibers can be seen than in the muscles with fibers with contraction knots seen in Figure 2 (initial magnification: 200x). (b) After a more powerful contraction by immersing the muscles in formalin. The needle separates most of the fibers and only few of them are sectioned. Arrowheads, muscle fiber injury points. Bracket, area with various lesions (initial magnification: 200x).

In order to achieve a sustained contraction, KCl has been used as a depolarizing agent and CaCl_2_ has been used to increase extracellular calcium and facilitate the contraction. Few seconds after the exposure of the muscle to this situation, a sustained muscle contraction is obtained. Those muscles are a model of human muscles with hypertonia. We observed more injured fibers in these muscles than in muscles where several contraction knots have been induced (Figure 3(a)). In this experimental situation, the highest number of fibers injured by the insertion of the needle was obtained (around 5; Table 1). However, the observed injury is less than expected.

### 3.4. DN in Muscles Treated with Formalin

Immersing the living muscles in formalin resulted in a powerful contraction. When DN was performed, most of the fibers were separated and few were sectioned (Figure 3(b)). Surprisingly, in muscles that mimic spastic muscles, the number of fibers injured by the needle was 30% less than in healthy muscles (Table 1).

### 3.5. *Ex vivo* Dry Needling Seen by Scanning Electron Microscopy (SEM)

**Figure 4:**
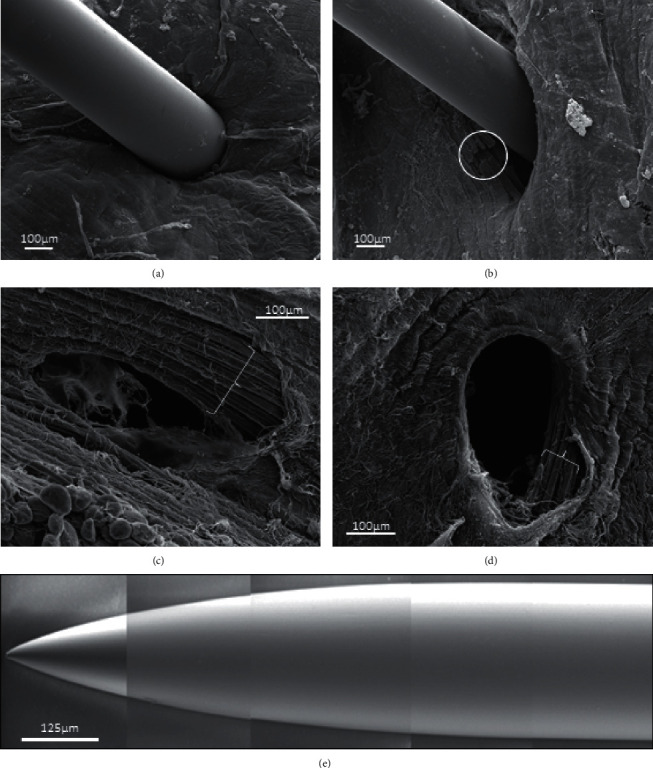
Scanning electron microscopy: examples of ex vivo dry needling in gastrocnemius muscle. The samples were processed without using collagenase. For this reason, the muscle fibers are mostly covered by endomysium and perimysium. This situation is especially evident in (a), where the needle punches the endomysium and perimysium. (b) A few injured muscle fibers are shown (white circle). Immediately below these, the fibers are unchanged. When the needle is removed, the muscle fibers return to its original position and the hole is almost closed (c), (d). Brackets, healthy muscle fibers. (e) Example of the tips of the needles used in DN.

To confirm the observations made by optical study with methylene blue on the LAL muscle, gastrocnemius muscles with contraction knots were treated with DN and studied with SEM. The SEM technique enables us to observe the tissue as a whole, and the muscle fibers were almost completely (Figure 4(a)) covered by connective tissue (Figure 4(b)). As observed by optical microscopy in the LAL muscle, only some muscle fibers were injured (Figure 4(b)) in gastrocnemius. Moreover, when the needle is removed, muscle fibers return back, and the hole is almost closed (Figures 4(c) and 4(d)). The tips of the DN needles used in this study are neither sharp nor beveled (Figure 4(e)).

## 4. Discussion

In order to analyze the effect of the repetitive mechanical injury induced by puncture in the muscle fiber at different degrees of contraction, we studied structural changes after 15 repeated punctures in the LAL muscle. Four conditions, healthy normal muscles, muscles with fibers, and contraction knots as well as chemically contracted muscles (CaCl_2_/KCl and formalin) have been studied. In most cases, the muscle fibers are not injured; instead, they are pushed away by the tip of the needle. The muscles manipulated in these experimental situations have preserved the ability to contract. It is known that injury to muscle fibers causes them to contract [22].

DN consists of the insertion of a solid intramuscular needle to eliminate or inactivate the MTrP. This inactivation requires the muscular injury, inflammatory reaction, and muscle regeneration [10]. In addition, multiple insertions are necessary to obtain a clinical benefit [7, 23, 24]. The most common number of punctures in patients is 8 to 10 insertions per intervention [8, 9]. However, there is no consensus on how many insertions are necessary to obtain clinical improvement. Some authors associate the success of DN with the generation of LTR. On the contrary, Fernández-Carnero et al. [16] in a randomized, double-blind clinical trial reported that different numbers of DN with and without RTL in the upper trapezius produce similar improvements in patients with cervical PG. A small lesion area will facilitate good muscle regeneration [23]. In the present study, we observed that the injury produced by the DN technique is small. In addition, to a small puncture hole, few fibers were injured, while the rest of fibers are only displaced by the passage of the tip of the needle. Healthy muscles are very elastic and upon the penetration of needles, fibers are displaced. In our laboratory, Domingo et al. have shown that there is complete recovery seven days after the injury by dry needling on mice skeletal muscle [25]. In the present study, we described that the damage caused by each insertion is limited to few muscle fibers. Furthermore, in the study by Domingo et al., repeated insertions (15 in total) were made to produce enough injured fibers in order to evaluate their regeneration. In such conditions, Domingo et al. also described that vascular injury occurs only sporadically [25]. The presence of vascular injury can imply poor irrigation of the injured area, which in turn makes difficult the muscular regeneration [26]. In the present study, we observed that the DN needle can frequently displace the blood vessels without damaging them. Thus, the vascular injury is minimal, which favors a good muscular regeneration in case of lesion.

Since MTrPs are placed in the innervation band [5], there is relevance to determine whether the DN technique could cause any damage to the intramuscular nerves. In the present study, similarly, as observed for blood vessels, intramuscular nerves are frequently displaced by the tip of the needle. Although Domingo et al. described that after DN distal nerve injury on normal muscles of mice can occur, no intramuscular nerve trunk lesions were evidenced [25]. To the best of our knowledge, there are no previous studies evaluating DN in muscle fibers with contraction knots.

Thirty minutes after a single subcutaneous injection of neostigmine (NTG), rodent muscles showed MTrPs [24]. In the present study, we provide evidence supporting the fact that the DN technique produced minimal lesions to healthy muscles. However, when the muscle tension is increased due to the presence of MTrPs, the lesion is greater. Treatment with neostigmine causes subsynaptic contraction knots to appear in the muscle fibers. It has been suggested that the shortening of the sarcomeres in contraction knots results in shortening of the muscle fiber and the generation of the taut band, therefore resulting in an increase in tension [22, 27, 28]. When the needle penetrates healthy areas of the muscle in the direction of the MTrPs, it does not cause a great injury as may be suggested. However, when the needle arrives in the area of the MTrPs, it is more effective in causing injury and affects the structure of the MTrPs. However, the level of injury is below expectation and more than one puncture is needed to achieve clinical results. In this regard, several years ago, Janet Travell developed a specific technique of DN that consisted of multiple insertions [7, 9, 21]. Dry needling is believed to damage or destroy the dysfunctional motor endplates of the MTrP [29]. The present work shows a low level of muscle and nerve injury produced by DN, indicating that the use of multiple insertions in the muscles of patients may be beneficial.

After a stroke, hypertonia or muscle spasticity is likely to appear [17]. DN is also performed in these muscles (DN for hypertonia and spasticity, DNHS®) [30]. Some studies tried to quantify the effects of the DNHS technique on the contractile properties of spastic muscles and a decrease in spasticity [20, 31] and an increase in active range of motion was reported [32, 33]. In order to increase muscle tone, we used normal Ringer rich in KCl and CaCl_2_. The first depolarizes the muscular and axonal membranes and the calcium enabled the increase of the neurotransmission and the availability of calcium for the contraction, thereby creating an artificial chemical model of contraction that was maintained over time. Hodgkin and Horowicz [34, 35] have previously described the quantitative effects of sudden changes in potassium concentration in the membrane potential and the effects produced on the contraction of the individual muscle fibers. In addition, Luettgau [36], working with external calcium, described that the process that controls the development of the tone of the muscle is influenced by the addition of solutions with high potassium content. In this model of hypertonia, the muscle tension is stronger than in the two previous situations. Possibly due to the greater sustained contraction, the muscles are stiffer and less flexible and therefore do not move as easily. In this model of hypertonia, DN was more harmful than muscles with fibers with contraction knots. Most of the muscle fibers were separated and only some were injured. The solid needle used may have a point blunt enough to push back the hypertonic fibers.

Unlike expected, the fibers of the fixed muscles suffered less injury than the other studied conditions. Fixation with formaldehyde is a crucial step to preserve cellular architecture and composition of cells in the tissue; in this case, the proteins are preserved. Several mechanisms can intervene in proteins fixation. It consists of a physical-chemical change of the molecule without altering the percentage of its constituent elements. They have more facility to be decomposed or digested but, unlike what is expected, the fibers that compose are less injured by the mechanical effect of the needle. Prior to denaturation, the proteins undergo a specific coagulation [37]. This process does not occur in a local area but appears in the whole set of fibers that are affected by formaldehyde and enables the separation of these fibers jointly when the needle is introduced.

In the SEM images, it was observed how the connective tissue could preserve the muscle fibers from damage. The collagen fibrils in the endomysium change direction as the muscle fibers stretch [38]. During the stretching process, the circumferentially oriented collagen fibers are reoriented in the longitudinal direction, increasing the stiffness of the fiber. The endomysium is not capable of transmitting the tensile forces of the muscle fibers in the resting length of the sarcomere. However, it transmits contractile force between adjacent muscle fibers by translaminar shear through the thickness of the connective tissue [39, 40]. Thus, the connective tissue of the endomysium could increase the resistance of the muscle fibers as their contractile tension increases under the different experimental conditions used in this study.

The tips of the DN needles used in this study (Figures 1(b) and 4(e)) are not sharp enough to justify a large injury. In a previous study, we evidenced with scanning electron microscopy that the tips of the needles commonly used in DN are dull [41]. These needles did not improve during repetitive insertions of either the skin only or the skin and muscle in patients. Thus, DN with these solid needles does not justify extensive muscle injury.

These results suggest that when puncturing deep muscles, the needle will not excessively injure healthy muscles when it passes through. In addition, the probability of injuring vessels and nerve trunks is low. In other words, it is a safe technique. In all the experimental situations used in this work, the probability of injuring muscle fibers is low. Therefore, if the intention of the dry needling is to inactivate all the contraction nodes of the patients' PGMs, there is need to perform several insertions. This is also true for patients with hypertonic or spastic muscles.

## 5. Limitations

As there are no previous studies similar to the present one, we have not calculated the sample size a priori; however the samples used have been enough to obtain significant differences compared to control.

DN has been performed on isolated muscles ex vivo. A muscle in these conditions does not have upper layers or skin that modify its response to DN. Also, due to their isolated condition, these muscles are denervated, so only passive responses of the tissue can be evaluated.

Isolated hypertonic and spastic muscles have been achieved by playing with the degree of contraction using local chemicals. In patients, these muscles would respond to a superior nervous problem.

## 6. Conclusion

Dry needling induces a lateral displacement of the muscle fibers and causes little damage. The present work shows a low level of muscle and nerve injury produced by DN, indicating that the use of multiple insertions in the muscles of patients may be beneficial.

## Figures and Tables

**Table 1:** Number of fibers injured by puncture (LAL muscle).

	Healthy muscles	Muscles with MTrPs	Mimicking hypertonic muscles	Mimicking spastic muscles
Mean ± SD	4.2 ± 2.9	4.7 ± 1.7	5.6 ± 2.3	3.0 ± 1.1
*P*		0.008	<0.001	<0.001
*n* of holes	225	195	225	135
95% intervals	3.9851 4.4238	4.4913 4.9959	5.3894 5.9440	2.8730 3.1714

Data expressed as the average number of fibers injured per hole created by the insertion of the needle ± SD. Fifteen insertions per LAL muscle and stained with methylene blue. In parentheses are the numbers of holes evaluated from 15 healthy muscles, 13 muscles with fibers with contraction knots, 15 hypertonic muscles, and 9 spastic muscles. Muscles submerged in a depolarizing Ringer solution (KCl, 20 mM) rich in calcium (CaCl_2_, 5 mM) mimicking human muscles with hypertonia. Muscles submerged muscles in a Ringer solution with formalin (4%) mimicking spastic muscles. *P* values were obtained by the Kolmogorov–Smirnov test and corresponds to comparison with respect to healthy muscles values.

## Data Availability

All data used to support the findings of this study are included within the article (Table 1) and as supplementary material (Raw Date Table 1.xls).

## Supplementary Materials

Supplementary Materials Table 1. The number of muscles and number of fibers injured per orifice are shown for each type of experimental procedure and control.
