# Supplementary material for: Dry Needling Produces Mild Injuries Irrespective to Muscle Stiffness and Tension in Ex Vivo Mice Muscles
Source: Pain Res Manag. 2022 Jul 5;2022:8920252. doi: 10.1155/2022/8920252 (PMC9277173; doi:10.1155/2022/8920252)
Supplement: Supplementary Materials — Table 1. The number of muscles and number of fibers injured per orifice are shown for each type of experimental procedure and control. [file 8920252.f1.docx]

| Num healthy muscle | Num damaged fibers by hole |  |
| --- | --- | --- |
| 1 | 4 |  |
|  | 4 |  |
|  | 6 |  |
|  | 6 |  |
|  | 4 |  |
|  | 4 |  |
|  | 4 |  |
|  | 6 |  |
|  | 6 |  |
|  | 1 |  |
|  | 2 |  |
|  | 4 |  |
|  | 4 |  |
|  | 4 |  |
|  | 4 |  |
| 2 | 6 |  |
|  | 4 |  |
|  | 4 |  |
|  | 4 |  |
|  | 4 |  |
|  | 0 |  |
|  | 3 |  |
|  | 6 |  |
|  | 3 |  |
|  | 5 |  |
|  | 4 |  |
|  | 6 |  |
|  | 6 |  |
|  | 1 |  |
|  | 4 |  |
| 3 | 4 |  |
|  | 6 |  |
|  | 6 |  |
|  | 2 |  |
|  | 3 |  |
|  | 5 |  |
|  | 4 |  |
|  | 5 |  |
|  | 6 |  |
|  | 7 |  |
|  | 2 |  |
|  | 6 |  |
|  | 4 |  |
|  | 2 |  |
|  | 4 |  |
| 4 | 6 |  |
|  | 4 |  |
|  | 4 |  |
|  | 4 |  |
|  | 6 |  |
|  | 6 |  |
|  | 1 |  |
|  | 4 |  |
|  | 5 |  |
|  | 6 |  |
|  | 1 |  |
|  | 2 |  |
|  | 6 |  |
|  | 6 |  |
|  | 4 |  |
| 5 | 3 |  |
|  | 5 |  |
|  | 4 |  |
|  | 5 |  |
|  | 6 |  |
|  | 7 |  |
|  | 2 |  |
|  | 6 |  |
|  | 4 |  |
|  | 4 |  |
|  | 6 |  |
|  | 6 |  |
|  | 2 |  |
|  | 4 |  |
|  | 5 |  |
| 6 | 6 |  |
|  | 1 |  |
|  | 2 |  |
|  | 6 |  |
|  | 6 |  |
|  | 5 |  |
|  | 4 |  |
|  | 5 |  |
|  | 6 |  |
|  | 1 |  |
|  | 7 |  |
|  | 2 |  |
|  | 6 |  |
|  | 4 |  |
|  | 4 |  |
| 7 | 6 |  |
|  | 6 |  |
|  | 4 |  |
|  | 5 |  |
|  | 6 |  |
|  | 1 |  |
|  | 2 |  |
|  | 6 |  |
|  | 4 |  |
|  | 2 |  |
|  | 4 |  |
|  | 5 |  |
|  | 6 |  |
|  | 1 |  |
|  | 2 |  |
| 8 | 7 |  |
|  | 6 |  |
|  | 6 |  |
|  | 4 |  |
|  | 2 |  |
|  | 4 |  |
|  | 6 |  |
|  | 3 |  |
|  | 6 |  |
|  | 4 |  |
|  | 5 |  |
|  | 6 |  |
|  | 1 |  |
|  | 2 |  |
|  | 3 |  |
| 9 | 2 |  |
|  | 3 |  |
|  | 5 |  |
|  | 4 |  |
|  | 5 |  |
|  | 6 |  |
|  | 7 |  |
|  | 2 |  |
|  | 6 |  |
|  | 3 |  |
|  | 6 |  |
|  | 4 |  |
|  | 2 |  |
|  | 4 |  |
|  | 6 |  |
| 10 | 3 |  |
|  | 6 |  |
|  | 4 |  |
|  | 5 |  |
|  | 4 |  |
|  | 0 |  |
|  | 3 |  |
|  | 6 |  |
|  | 3 |  |
|  | 2 |  |
|  | 4 |  |
|  | 6 |  |
|  | 2 |  |
|  | 6 |  |
|  | 4 |  |
| 11 | 3 |  |
|  | 4 |  |
|  | 5 |  |
|  | 6 |  |
|  | 4 |  |
|  | 5 |  |
|  | 6 |  |
|  | 3 |  |
|  | 5 |  |
|  | 2 |  |
|  | 6 |  |
|  | 4 |  |
|  | 2 |  |
|  | 4 |  |
|  | 0 |  |
| 12 | 3 |  |
|  | 6 |  |
|  | 3 |  |
|  | 5 |  |
|  | 4 |  |
|  | 6 |  |
|  | 6 |  |
|  | 2 |  |
|  | 6 |  |
|  | 4 |  |
|  | 5 |  |
|  | 4 |  |
|  | 5 |  |
|  | 6 |  |
|  | 1 |  |
| 13 | 3 |  |
|  | 2 |  |
|  | 6 |  |
|  | 4 |  |
|  | 5 |  |
|  | 6 |  |
|  | 1 |  |
|  | 2 |  |
|  | 6 |  |
|  | 4 |  |
|  | 2 |  |
|  | 4 |  |
|  | 5 |  |
|  | 3 |  |
|  | 6 |  |
| 14 | 4 |  |
|  | 2 |  |
|  | 4 |  |
|  | 6 |  |
|  | 3 |  |
|  | 6 |  |
|  | 4 |  |
|  | 5 |  |
|  | 4 |  |
|  | 1 |  |
|  | 3 |  |
|  | 6 |  |
|  | 2 |  |
|  | 4 |  |
|  | 6 |  |
| 15 | 3 |  |
|  | 6 |  |
|  | 4 |  |
|  | 5 |  |
|  | 4 |  |
|  | 5 |  |
|  | 6 |  |
|  | 1 |  |
|  | 6 |  |
|  | 3 |  |
|  | 5 |  |
|  | 4 |  |
|  | 6 |  |
|  | 6 |  |
|  | 2 |  |

| Num muscle with contraction knots | Num damaged fibers by hole |  |
| --- | --- | --- |
| 1 | 3 |  |
|  | 7 |  |
|  | 5 |  |
|  | 4 |  |
|  | 8 |  |
|  | 2 |  |
|  | 7 |  |
|  | 4 |  |
|  | 3 |  |
|  | 4 |  |
|  | 7 |  |
|  | 8 |  |
|  | 4 |  |
|  | 5 |  |
|  | 4 |  |
| 2 | 4 |  |
|  | 3 |  |
|  | 3 |  |
|  | 6 |  |
|  | 3 |  |
|  | 5 |  |
|  | 4 |  |
|  | 5 |  |
|  | 8 |  |
|  | 3 |  |
|  | 4 |  |
|  | 4 |  |
|  | 6 |  |
|  | 6 |  |
|  | 8 |  |
| 3 | 3 |  |
|  | 3 |  |
|  | 4 |  |
|  | 5 |  |
|  | 6 |  |
|  | 8 |  |
|  | 4 |  |
|  | 6 |  |
|  | 4 |  |
|  | 3 |  |
|  | 4 |  |
|  | 8 |  |
|  | 4 |  |
|  | 3 |  |
|  | 4 |  |
| 4 | 6 |  |
|  | 8 |  |
|  | 3 |  |
|  | 4 |  |
|  | 5 |  |
|  | 6 |  |
|  | 8 |  |
|  | 3 |  |
|  | 6 |  |
|  | 3 |  |
|  | 4 |  |
|  | 3 |  |
|  | 5 |  |
|  | 4 |  |
|  | 5 |  |
| 5 | 2 |  |
|  | 7 |  |
|  | 3 |  |
|  | 3 |  |
|  | 4 |  |
|  | 3 |  |
|  | 6 |  |
|  | 5 |  |
|  | 8 |  |
|  | 4 |  |
|  | 5 |  |
|  | 6 |  |
|  | 8 |  |
|  | 3 |  |
|  | 6 |  |
| 6 | 3 |  |
|  | 5 |  |
|  | 4 |  |
|  | 5 |  |
|  | 6 |  |
|  | 3 |  |
|  | 8 |  |
|  | 4 |  |
|  | 7 |  |
|  | 4 |  |
|  | 4 |  |
|  | 8 |  |
|  | 4 |  |
|  | 4 |  |
|  | 5 |  |
| 7 | 6 |  |
|  | 7 |  |
|  | 3 |  |
|  | 8 |  |
|  | 3 |  |
|  | 3 |  |
|  | 4 |  |
|  | 5 |  |
|  | 6 |  |
|  | 4 |  |
|  | 2 |  |
|  | 3 |  |
|  | 6 |  |
|  | 8 |  |
|  | 4 |  |
| 8 | 3 |  |
|  | 4 |  |
|  | 8 |  |
|  | 3 |  |
|  | 4 |  |
|  | 3 |  |
|  | 5 |  |
|  | 6 |  |
|  | 7 |  |
|  | 2 |  |
|  | 3 |  |
|  | 4 |  |
|  | 3 |  |
|  | 8 |  |
|  | 4 |  |
| 9 | 5 |  |
|  | 8 |  |
|  | 8 |  |
|  | 3 |  |
|  | 8 |  |
|  | 3 |  |
|  | 6 |  |
|  | 4 |  |
|  | 3 |  |
|  | 4 |  |
|  | 6 |  |
|  | 3 |  |
|  | 6 |  |
|  | 4 |  |
|  | 5 |  |
| 10 | 6 |  |
|  | 6 |  |
|  | 3 |  |
|  | 4 |  |
|  | 7 |  |
|  | 6 |  |
|  | 8 |  |
|  | 4 |  |
|  | 7 |  |
|  | 3 |  |
|  | 8 |  |
|  | 2 |  |
|  | 3 |  |
|  | 4 |  |
|  | 5 |  |
| 11 | 2 |  |
|  | 4 |  |
|  | 3 |  |
|  | 8 |  |
|  | 3 |  |
|  | 2 |  |
|  | 4 |  |
|  | 8 |  |
|  | 3 |  |
|  | 6 |  |
|  | 4 |  |
|  | 3 |  |
|  | 4 |  |
|  | 5 |  |
|  | 6 |  |
| 12 | 4 |  |
|  | 5 |  |
|  | 8 |  |
|  | 3 |  |
|  | 5 |  |
|  | 2 |  |
|  | 8 |  |
|  | 4 |  |
|  | 4 |  |
|  | 4 |  |
|  | 3 |  |
|  | 3 |  |
|  | 6 |  |
|  | 3 |  |
|  | 6 |  |
| 13 | 4 |  |
|  | 3 |  |
|  | 4 |  |
|  | 6 |  |
|  | 3 |  |
|  | 8 |  |
|  | 4 |  |
|  | 3 |  |
|  | 2 |  |
|  | 4 |  |
|  | 4 |  |
|  | 8 |  |
|  | 4 |  |
|  | 5 |  |
|  | 3 |  |
|  |  |  |

| Num mimicking hypertonic muscle | Num damaged fibers by hole |  |
| --- | --- | --- |
| 1 | 8 |  |
|  | 5 |  |
|  | 4 |  |
|  | 4 |  |
|  | 5 |  |
|  | 5 |  |
|  | 4 |  |
|  | 9 |  |
|  | 5 |  |
|  | 3 |  |
|  | 8 |  |
|  | 5 |  |
|  | 8 |  |
|  | 1 |  |
|  | 5 |  |
| 2 | 9 |  |
|  | 8 |  |
|  | 8 |  |
|  | 9 |  |
|  | 9 |  |
|  | 5 |  |
|  | 5 |  |
|  | 8 |  |
|  | 5 |  |
|  | 8 |  |
|  | 8 |  |
|  | 7 |  |
|  | 3 |  |
|  | 3 |  |
|  | 3 |  |
| 3 | 3 |  |
|  | 8 |  |
|  | 8 |  |
|  | 9 |  |
|  | 9 |  |
|  | 5 |  |
|  | 5 |  |
|  | 8 |  |
|  | 5 |  |
|  | 8 |  |
|  | 8 |  |
|  | 7 |  |
|  | 3 |  |
|  | 5 |  |
|  | 3 |  |
| 4 | 8 |  |
|  | 4 |  |
|  | 5 |  |
|  | 4 |  |
|  | 6 |  |
|  | 8 |  |
|  | 9 |  |
|  | 4 |  |
|  | 5 |  |
|  | 6 |  |
|  | 9 |  |
|  | 4 |  |
|  | 6 |  |
|  | 8 |  |
|  | 4 |  |
| 5 | 3 |  |
|  | 5 |  |
|  | 4 |  |
|  | 5 |  |
|  | 6 |  |
|  | 7 |  |
|  | 9 |  |
|  | 8 |  |
|  | 4 |  |
|  | 4 |  |
|  | 9 |  |
|  | 8 |  |
|  | 9 |  |
|  | 4 |  |
|  | 5 |  |
| 6 | 6 |  |
|  | 8 |  |
|  | 2 |  |
|  | 6 |  |
|  | 9 |  |
|  | 5 |  |
|  | 4 |  |
|  | 5 |  |
|  | 6 |  |
|  | 9 |  |
|  | 7 |  |
|  | 6 |  |
|  | 6 |  |
|  | 5 |  |
|  | 4 |  |
| 7 | 6 |  |
|  | 9 |  |
|  | 4 |  |
|  | 5 |  |
|  | 6 |  |
|  | 8 |  |
|  | 5 |  |
|  | 6 |  |
|  | 4 |  |
|  | 8 |  |
|  | 4 |  |
|  | 5 |  |
|  | 6 |  |
|  | 7 |  |
|  | 2 |  |
| 8 | 9 |  |
|  | 6 |  |
|  | 7 |  |
|  | 4 |  |
|  | 2 |  |
|  | 4 |  |
|  | 6 |  |
|  | 3 |  |
|  | 6 |  |
|  | 4 |  |
|  | 5 |  |
|  | 8 |  |
|  | 9 |  |
|  | 9 |  |
|  | 3 |  |
| 9 | 2 |  |
|  | 8 |  |
|  | 5 |  |
|  | 4 |  |
|  | 5 |  |
|  | 6 |  |
|  | 9 |  |
|  | 9 |  |
|  | 6 |  |
|  | 3 |  |
|  | 6 |  |
|  | 4 |  |
|  | 2 |  |
|  | 4 |  |
|  | 6 |  |
| 10 | 3 |  |
|  | 5 |  |
|  | 8 |  |
|  | 9 |  |
|  | 4 |  |
|  | 5 |  |
|  | 4 |  |
|  | 5 |  |
|  | 4 |  |
|  | 5 |  |
|  | 9 |  |
|  | 4 |  |
|  | 5 |  |
|  | 6 |  |
|  | 8 |  |
| 11 | 3 |  |
|  | 8 |  |
|  | 9 |  |
|  | 5 |  |
|  | 4 |  |
|  | 9 |  |
|  | 3 |  |
|  | 6 |  |
|  | 3 |  |
|  | 2 |  |
|  | 4 |  |
|  | 6 |  |
|  | 8 |  |
|  | 6 |  |
|  | 4 |  |
| 12 | 3 |  |
|  | 4 |  |
|  | 5 |  |
|  | 9 |  |
|  | 4 |  |
|  | 5 |  |
|  | 6 |  |
|  | 8 |  |
|  | 5 |  |
|  | 8 |  |
|  | 9 |  |
|  | 4 |  |
|  | 5 |  |
|  | 4 |  |
|  | 5 |  |
| 13 | 3 |  |
|  | 6 |  |
|  | 3 |  |
|  | 5 |  |
|  | 4 |  |
|  | 8 |  |
|  | 7 |  |
|  | 2 |  |
|  | 6 |  |
|  | 4 |  |
|  | 5 |  |
|  | 4 |  |
|  | 5 |  |
|  | 6 |  |
|  | 9 |  |
| 14 | 9 |  |
|  | 2 |  |
|  | 7 |  |
|  | 9 |  |
|  | 5 |  |
|  | 6 |  |
|  | 8 |  |
|  | 5 |  |
|  | 6 |  |
|  | 4 |  |
|  | 2 |  |
|  | 4 |  |
|  | 5 |  |
|  | 3 |  |
|  | 9 |  |
| 15 | 4 |  |
|  | 2 |  |
|  | 4 |  |
|  | 9 |  |
|  | 3 |  |
|  | 6 |  |
|  | 9 |  |
|  | 5 |  |
|  | 8 |  |
|  | 5 |  |
|  | 3 |  |
|  | 8 |  |
|  | 2 |  |
|  | 4 |  |
|  | 6 |  |

| Num mimicking spastic muscle | Num damaged fibers by hole |  |
| --- | --- | --- |
| 1 | 4 |  |
|  | 2 |  |
|  | 3 |  |
|  | 2 |  |
|  | 3 |  |
|  | 2 |  |
|  | 4 |  |
|  | 2 |  |
|  | 3 |  |
|  | 4 |  |
|  | 2 |  |
|  | 4 |  |
|  | 2 |  |
|  | 3 |  |
|  | 4 |  |
| 2 | 3 |  |
|  | 1 |  |
|  | 4 |  |
|  | 2 |  |
|  | 4 |  |
|  | 2 |  |
|  | 3 |  |
|  | 4 |  |
|  | 3 |  |
|  | 4 |  |
|  | 4 |  |
|  | 3 |  |
|  | 3 |  |
|  | 2 |  |
|  | 4 |  |
| 3 | 4 |  |
|  | 3 |  |
|  | 4 |  |
|  | 3 |  |
|  | 3 |  |
|  | 4 |  |
|  | 4 |  |
|  | 2 |  |
|  | 2 |  |
|  | 2 |  |
|  | 2 |  |
|  | 4 |  |
|  | 2 |  |
|  | 3 |  |
|  | 4 |  |
| 4 | 2 |  |
|  | 4 |  |
|  | 4 |  |
|  | 4 |  |
|  | 2 |  |
|  | 2 |  |
|  | 3 |  |
|  | 4 |  |
|  | 3 |  |
|  | 2 |  |
|  | 3 |  |
|  | 2 |  |
|  | 2 |  |
|  | 2 |  |
|  | 4 |  |
| 5 | 3 |  |
|  | 4 |  |
|  | 3 |  |
|  | 4 |  |
|  | 2 |  |
|  | 4 |  |
|  | 2 |  |
|  | 3 |  |
|  | 4 |  |
|  | 4 |  |
|  | 3 |  |
|  | 3 |  |
|  | 2 |  |
|  | 4 |  |
|  | 2 |  |
| 6 | 4 |  |
|  | 3 |  |
|  | 2 |  |
|  | 3 |  |
|  | 2 |  |
|  | 4 |  |
|  | 4 |  |
|  | 3 |  |
|  | 2 |  |
|  | 2 |  |
|  | 3 |  |
|  | 2 |  |
|  | 3 |  |
|  | 4 |  |
|  | 4 |  |
| 7 | 3 |  |
|  | 4 |  |
|  | 1 |  |
|  | 2 |  |
|  | 3 |  |
|  | 2 |  |
|  | 4 |  |
|  | 3 |  |
|  | 4 |  |
|  | 2 |  |
|  | 4 |  |
|  | 3 |  |
|  | 4 |  |
|  | 3 |  |
|  | 2 |  |
| 8 | 4 |  |
|  | 2 |  |
|  | 3 |  |
|  | 4 |  |
|  | 2 |  |
|  | 4 |  |
|  | 3 |  |
|  | 3 |  |
|  | 4 |  |
|  | 4 |  |
|  | 4 |  |
|  | 2 |  |
|  | 3 |  |
|  | 2 |  |
|  | 3 |  |
| 9 | 2 |  |
|  | 3 |  |
|  | 2 |  |
|  | 4 |  |
|  | 2 |  |
|  | 4 |  |
|  | 2 |  |
|  | 4 |  |
|  | 4 |  |
|  | 2 |  |
|  | 4 |  |
|  | 4 |  |
|  | 2 |  |
|  | 4 |  |
|  | 3 |  |

First column: Number of muscles

Second column: Number of fibers injured by hole

Fifteen insertions per LAL muscle and stained with methylene blue.

N muscles= 15 healthy muscles, 13 muscles with fibers with contraction knots, 15 hypertonic muscles and 9 spastic muscles.

Muscles submerged in a depolarizing Ringer solution (KCl, 20mM) rich in calcium (CaCl2, 5mM) mimicking human muscles with hypertonia. Muscles submerged muscles in a Ringer solution with formalin (4%) mimicking spastic muscles.
